# A Web-Based Interactive Diabetes Registry for Health Care Management and Planning in Saudi Arabia

**DOI:** 10.2196/jmir.2722

**Published:** 2013-09-11

**Authors:** Khalid A Al-Rubeaan, Amira M Youssef, Shazia N Subhani, Najlaa A Ahmad, Ahmad H Al-Sharqawi, Heba M Ibrahim

**Affiliations:** ^1^Strategic Center for Diabetes ResearchUniversity Diabetes CentreKing Saud UniversityRiyadhSaudi Arabia; ^2^Registry DepartmentUniversity Diabetes CenterKing Saud UniversityRiyadhSaudi Arabia; ^3^Registries Core FacilityBiostatistics, Epidemiology and Scientific ComputingKing Faisal Specialist Hospital and Research CentreRiyadhSaudi Arabia; ^4^Biostatistics DepartmentUniversity Diabetes CenterKing Saud UniversityRiyadhSaudi Arabia

**Keywords:** diabetes mellitus, registries, geographic information systems, medical records systems, computerized, health planning, research report, data bank, factual, epidemiology

## Abstract

**Background:**

Worldwide, eHealth is a rapidly growing technology. It provides good quality health services at lower cost and increased availability. Diabetes has reached an epidemic stage in Saudi Arabia and has a medical and economic impact at a countrywide level. Data are greatly needed to better understand and plan to prevent and manage this medical problem.

**Objective:**

The Saudi National Diabetes Registry (SNDR) is an electronic medical file supported by clinical, investigational, and management data. It functions as a monitoring tool for medical, social, and cultural bases for primary and secondary prevention programs. Economic impact, in the form of direct or indirect cost, is part of the registry’s scope. The registry’s geographic information system (GIS) produces a variety of maps for diabetes and associated diseases. In addition to availability and distribution of health facilities in the Kingdom, GIS data provide health planners with the necessary information to make informed decisions. The electronic data bank serves as a research tool to help researchers for both prospective and retrospective studies.

**Methods:**

A Web-based interactive GIS system was designed to serve as an electronic medical file for diabetic patients retrieving data from medical files by trained registrars. Data was audited and cleaned before it was archived in the electronic filing system. It was then used to produce epidemiologic, economic, and geographic reports. A total of 84,942 patients were registered from 2000 to 2012, growing by 10% annually.

**Results:**

The SNDR reporting system for epidemiology data gives better understanding of the disease pattern, types, and gender characteristics. Part of the reporting system is to assess quality of health care using different parameters, such as HbA1c, that gives an impression of good diabetes control for each institute. Economic reports give accurate cost estimation of different services given to diabetic patients, such as the annual insulin cost per patient for type 1, type 2, and gestational diabetes, which are 1155 SR (US $308), 1406 SR (US $375), and 1002 SR (US $267), respectively. Of this, 72.02% of the total insulin cost is spent on type 2 patients and 55.39% is in the form of premixed insulin. The SNDR can provide an accurate assessment of the services provided for research purposes. For example, only 27.00% of registered patients had an ophthalmic examination and only 71.10% of patients with proliferative retinopathy had laser therapy.

**Conclusions:**

The SNDR is an effective electronic medical file that can provide epidemiologic, economic, and geographic reports that can be used for disease management and health care planning. It is a useful tool for research and disease health care quality monitoring.

## Introduction

The development of computerized applications and telecommunication for computer-based health care management tools has increased and helped patients, physicians, and health institutes better manage health and disease. Nowadays, eHealth is considered one of the most rapidly growing technologies worldwide. It aims to provide health services at a lower cost with good quality and availability. Chronic diseases are known for their high rate of morbidity, disability, and mortality, in addition to their high cost; they amounted to 75% of health care expenditures in the United States during the year 2000 [1]. Therefore, eHealth is expected to reduce the effect of such diseases on health and economy. Diabetes mellitus is the most important and frequent chronic disease, as reported by the International Diabetes Federation (IDF), with more than 366 million people suffering from this disease worldwide and the number likely to be 552 million by 2030 [2].

Diabetes morbidity is related to chronic complications, namely neuropathy, nephropathy, retinopathy, and vasculopathy making it the leading cause of blindness, renal failure, and lower limb amputation. The prevalence of mild to severe diabetic neuropathy ranges from 60% to 70% [3]. In 2004, more than 60% of nontraumatic lower limb amputations were related to diabetes [4]. Among both diagnosed and undiagnosed diabetic patients, the prevalence of retinopathy ranges from 17.6% to 33.2% [5], whereas the prevalence rate for vision-threatening retinopathy was 8.2% [6]. Coronary heart disease prevalence reported among adult diabetic patients was as high as 55% [7]. The prevalence of diabetic nephropathy among type 2 diabetic patients ranges from 7.6% to 55% [8].

Deaths attributed to diabetes globally increased by 5.5% in the year 2010 compared to the year 2007 [9]. This increase is largely because of a 29% increase in the number of deaths in North America and the Caribbean region, but also a 12% increase in the Southeast Asia region and an 11% increase in the Western Pacific region [10]. Diabetes is also known to be a leading cause of death largely because of increased risk of coronary artery disease and stroke. According to World Health Organization (WHO) data, more than 75% of patients with non-insulin-dependent diabetes mellitus die because of vascular accidents [11].

Saudi Arabia is considered to be 1 of the top 10 countries in terms of diabetes prevalence worldwide [12]. Diabetes prevalence has been estimated at 23.7%, and varies according to the geographic region of the Kingdom, being the highest in the Northern and Eastern regions, which account for 27.9% and 26.4% of cases, respectively. The Western and Central regions were 24.7% and 23.7%, respectively. The lowest prevalence is in the Southern region, which accounts for 18.2% [13].

The prevalence of diabetes chronic complications in Saudi Arabia has also been considered to be one of the highest worldwide, with 82% for neuropathy [14], 31% for retinopathy [15], and 32.1% for nephropathy [16]. Diabetes in Saudi Arabia has been found to be responsible for 30% to 45% of patients requiring dialysis [17], and 37% to 41% of patients with stroke [18].

From the currently available data on diabetes and its complications, it is very clear that this disease has reached an epidemic stage and has a medical and economic impact on health and economy of the Kingdom. This is associated with deficiency in the data required for proper action to prevent and manage this huge medical problem. In spite of the good health system and facilities currently available in the Kingdom, health care provided to diabetic patients has fallen short of achieving optimal clinical outcomes. This can be attributed to the large number of patients and the limited time allotted for each patient, in which new technology can contribute for good patient’s monitoring and high level of clinical practice. Thus, using a diabetes registry can give us a better understanding of the disease and its impact on patients and the health system. It also provides a chance for research and better planning for disease management in setting the proper standards for medical care. Eventually, it could provide physicians with feedback on their medical care, guiding them to improve their clinical outcomes [19]. It also serves as the basis for epidemiology data, providing better insight into diabetes complications and associated diseases, and aiming to improve disease management and health care quality.

Disease registries currently available cover a wide spectrum of conditions, such as infectious diseases, cancers, congenital diseases, and rare diseases, such as cystic fibrosis. Chronic illnesses, such as diabetes, heart failure, end-stage renal disease, myocardial infarction, or stroke, have been the target for disease registries in many countries. A survey of 1040 US physician organizations showed that diabetes registries are used 40.3% of the time, asthma registries 31.2%, congestive heart failure registries 34.8%, and depression registries 15.7% [20]. There are limited numbers of diabetes registries globally, some of which are brief and disease-focused, whereas others are made to serve certain objectives. On the other hand, there is a third group of registries made to serve diabetic patients in hospital settings, or that considers diabetes as a component of chronic diseases. Joslin’s Web-based Diabetes Registry and Risk Stratification System is a Web-based application using Joslin’s evidence-based Clinical Guidelines to identify and intervene with patients who are most likely to develop costly, debilitating, diabetes-related complications [21]. On the other hand, Penn State Hershey Diabetes and Obesity Institute Registry (PSHDOI) is a custom-built application that assists in tracking clinical outcomes for diabetic patients [22]. The Chronic Disease Electronic Management System (CDEMS) has embedded guidelines for a variety of chronic diseases (diabetes, atrial fibrillation, heart failure, coronary heart disease, hyperlipidemia, depression, asthma, and osteoporosis) [23]. None of the currently available registries has used a diabetes registry in a holistic approach or utilized geographic mapping and economic assessment of the disease countrywide.

The Saudi National Diabetes Registry (SNDR) was established with the primary goal of developing a database for diagnosed national diabetic patients living in the Kingdom of Saudi Arabia. The SNDR’s objective is to function as an electronic medical file to provide medical teams with clinical, investigational, and management information. It also functions as a surveillance-monitoring tool for clinical and epidemiology practitioners by providing key performance indicators related to this disease in either acute or chronic circumstances. The SNDR will provide data related to the association of diabetes with hypertension, hyperlipidemia, and obesity.

Assessing the economic impact of this disease in the form of direct and indirect costs is part of the SNDR objectives. Social and cultural variables are used by the SNDR system to help in planning for primary and secondary prevention programs. Health facilities and management tool availability reports are produced periodically, which give health planners clear insights and invite proper solutions to be found. The SNDR acts as an advisory body for different heath regions by coordinating data, knowledge, and plans about diabetes and related medical conditions to both national and international institutes.

The registry uses a geographic information system (GIS) with its environmental correlation to produce a variety of maps and reports focusing on diabetes and associated diseases in different health regions. It will also map health care institutions and medical facilities availability and distribution in the Kingdom.

In this paper, an overview of the SNDR structure, functionalities, and reporting system is discussed, and different examples from the reporting systems are given.

## Methods

The SNDR is a national government-funded project located in Riyadh, the capital of Saudi Arabia. The program began in1997 with hard copy registry files, which were converted into an electronic Web-based system in 2000. The design and development of the Web-based SNDR has been explained in a previously published article [24].

The registry includes both governmental and private hospitals in addition to primary health care centers. Based on reviews of hospital medical records, highly trained full-time data registrars are assigned to each health institute after an intensive training course on the diabetes registry.

Saudi patients with any type of diabetes, regardless of their age or gender, are eligible for the SNDR. The National Identification Number is used as a unique identifier to avoid any form of duplication. Case classification is performed using American Diabetes Association (ADA) criteria, which designate patients as type 1, type 2, impaired glucose tolerance (IGT), gestational diabetes mellitus (GDM), and secondary diabetes.

The patient’s clinical data collection form includes the patient’s name, residence location, complete contact details, date of birth, and marital status. Detailed diabetes history includes diabetes type, date of diagnosis, and associated diseases. Social history, including smoking, educational level, occupation, and income, is retrieved from the patient’s file. Clinical parameters included are height, weight, and waist circumference. In addition to blood pressure and glycemic markers, fasting blood sugar, random blood sugar, 2-hour post-meal blood sugar, and glycated hemoglobin (HbA1c) are also collected. Laboratory measurements include urine analyses for glucose, protein, ketones, liver enzymes assessment, including alkaline phosphate, serum glutamic-pyruvic transaminase (SGPT), serum glutamic oxaloacetic transaminase (SGOT), and total protein; thyroid function test, including thyroid-stimulating hormone (TSH), T4, and T3; and lipids profile, including cholesterol, triglycerides, high-density lipoprotein (HDL), and low-density lipoprotein (LDL). Lifestyle related to diet and exercise and different therapeutic modalities, namely insulin and oral agents are also included in the registry file. The registry file includes chronic complications, including neuropathy, retinopathy, nephropathy, and vasculopathy, in addition to any associated diseases, such as hypertension, hyperlipidemia, thyroid disease, and others.

Both institutional and national auditing systems are adopted by the SNDR to ascertain data. Approximately 10% to 15% of the registry hard copy files from each institution are randomly selected by the institutional auditor for this purpose. All hard copy registry forms are archived in the national registry archiving room. Each data encoder uses a password-locked access code for data encoding in the registry Web-based software program. National data auditing, cleaning, and validation are performed by a well-trained national auditor, and all soft copy registry data form the data bank for the SNDR. The data bank has a very strong, secure system that protects data from viruses and hackers, as explained in detail in a previous publication [24].

The SNDR has a functionality to query maps by using GIS (Esri, Redlands, CA, USA) consisting of ArcGIS server and desktop ArcGIS version 10 for the design and publishing of all maps. For all designed maps, the World Geodetic System 1984 (WGS1984) geographic coordinate system (GCS) was used. Initial projection scale was 1:10,000,000. The data source for population and city coordinates was the Ministry of Planning and Ministry of Defense using the year 1428 hijri, representing the year 2007 data statistics [25]. Regional gradation on the maps is a representation of the Saudi population at the regional level. The point symbology is a representation of the patient count from various cities. Hospital locations/coordinates were identified using Google Earth [26].

A customized statistical reporting system was used to produce different epidemiology tables and graphs. To input accurate data into this registry, the SNDR registry data are linked to the government citizen database, through the main electronic portal for government and financial sectors [27] that provides additional information that can be used for social and cultural studies.

**Figure 1:**
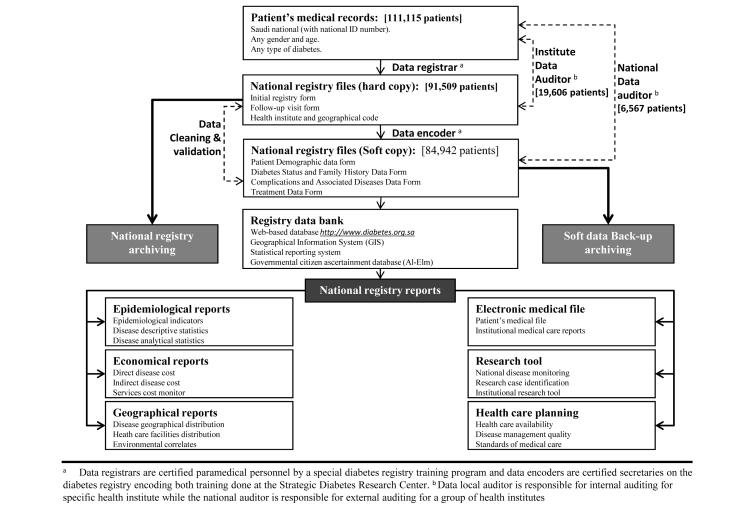
The Saudi National Diabetes Registry structure of data collection, encoding, auditing, and archiving plus its reporting systems.

Figure 1 demonstrates the structure for data collection, encoding, auditing, archiving, and the reporting system for the SNDR. National auditors are responsible for national data auditing, and continuous active cleaning and validation of patients’ files before the soft copy file is archived electronically in the SNDR data bank. A highly specialized bioinformatics and statistical team is in charge of producing national registry reports, which include epidemiology, economic, and geographic reports. At the same time, an information technologist and public health specialist are responsible for upgrading the system and preparing reports, which can be used for disease surveillance and public health care planning.

The epidemiology reports generated by the registry include epidemiology indicators and both descriptive and analytical statistics, in addition to providing economic reports that give both direct and indirect disease cost based on the local cost estimate and specific cost analyses for different services and disease management processes. GIS reports provide a geographic description for disease and its chronic complications or associated disease spatial distribution and their environmental correlations. The geographic distribution of health care facilities and management availabilities can help stakeholders better understand the disease and detect gaps in continuum of care, such as lack of facilities or personnel.

The national registry database functions as a patient’s medical file, and it is used by the institutions for the same purpose. The plan is to allow all registered patients to access their own files through their national ID number, and to allow them to print out their latest medical report. National disease monitoring and research case identification will be part of the SNDR to encourage researchers in this field at the national or institutional level. The SNDR will be helpful to health care planners through its regular reports on health care availability and disease management quality. For this reason, the SNDR sets the standards of medical care provided for diabetic patients in this country. The main objective of SNDR is to provide different reports that will be useful to health providers, scientists, economists, researchers, and health care planners.

## Results

### Overview

**Figure 2:**
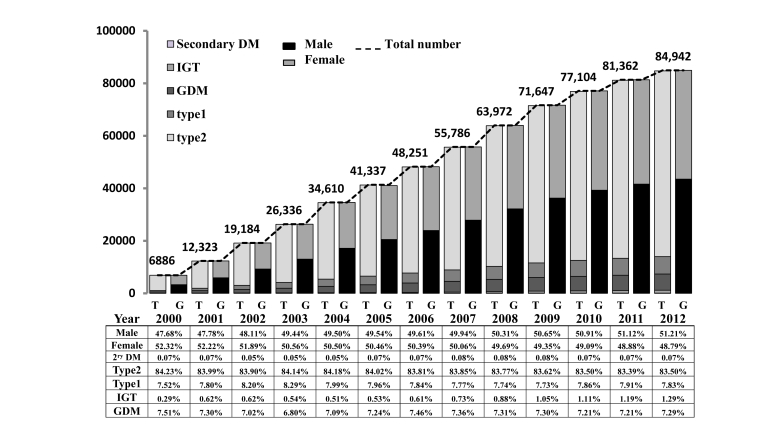
The yearly total number of registered cases of diabetes according to gender (G) and type (T) of diabetes from the start of registry in 2000 to 2012.

The SNDR currently hosts data on 111,115 patients, of which 19,606 are currently audited by institutional auditors and 6567 are in the process of national auditing at the time of this paper preparation. A total of 84,942 patients have passed data auditing, cleaning, and verification and were used to test the national registry reporting system. Figure 2 demonstrates the growth of the SNDR population over the past 12 years from 6886 Saudi diabetics registered in 2000 to 84,942 patients audited at the end of 2012. The registry is growing by 10% annually. The gender distribution is higher in males, accounting for 51.10% in 2012. The distribution of diabetes types was almost identical each of the 12 years; distribution in the year 2012 was 7.83%, 83.50%, 1.29%, 7.29%, and 0.07% for type 1, type 2, IGT, GDM, and secondary diabetes, respectively. The IGT cases are increasing with time from 0.29% in 2000 to 1.29% in 2012.

### Epidemiology Reports

Table 1 presents a descriptive and analytical statistical report of data from 22 randomly selected hospitals. The data show the total number of diabetic patients, gender, and diabetes type distribution in addition to mean HbA1c for comparative assessment. The number of diabetic patients varies in some hospitals from more than 10,000 diabetic patients to less than 200. The variation is seen in gender distribution, in which males are more numerous than females in some hospitals, but it is the opposite for others. The distribution of diabetes types is representative of the national distribution pattern except for the facility with smaller number of patients (301023). Type 2 diabetes is highly prevalent, ranging between 7.18% and 94.70%, followed by type 1 diabetes (ranges from 4.42% to 77.44%). The range of GDM varies widely, between 0.04% and 37.24%, which is also the same for IGT cases ranging from 0% to 5.56%. The mean HbA1c, an indicator for patients’ diabetes control and medical care provided in each institute, has a variable range between 7.3% and 13.6%.

### Economic Reports

Table 2 shows an annual economic report on consumption and cost distribution of different insulin types according to diabetes type for a total of 30,414 insulin-using patients. Insulin users represented 35.81% of total registered patients. Among insulin users, 26.49%, 67.34%, and 6.16% are type 1, type 2, and GDM, respectively. The annual average insulin costs according to diabetes type in Saudi Riyals (SR) are 1155 SR (US $308), 1406 SR (US $375), and 1002 SR (US $267) for type 1, type 2, and GDM, respectively. The total cost of insulin therapy is 39,996,370 SR (US $10,665,699); 23.28% is spent on type 1, whereas 72.03% and 4.70% are spent on type 2 and GDM, respectively. Premixed insulin contributes to 55.39% of the total insulin cost per year, regular insulin was 14.49%, and 12.54% was neutral protamine Hagedorn (NPH). The insulin analogs annual costs were 13.47%, 3.82%, and 0.28% for Glargine, Aspart, and Lispro, respectively. Each type of insulin was used more often with type 2 diabetic patients, especially premixed, with the exception of Aspart and Lispro, which were used more often with type 1 diabetic patients.

**Table 1:** Number of patients and frequency distribution from 22 randomly selected health care institutes according to gender, diabetes type, and mean HbA1c values.

Health institute code	Total patients, n (%) (N=84,467)	Number of patients according to gender, n (%)		Number of patients according to diabetes type, n (%)				Mean HbA1c (%)^a^
		Male (n=43,261)	Female (n=41,206)	Type 1 (n=6624)	Type 2 (n=70,293)	IGT^b^(n=1096)	GDM^c^(n=6195)	
301003	16,308 (19.31)	8016 (49.15)	8292 (50.85)	1131 (6.98)	12,897 (79.58)	220 (1.36)	1959 (12.09)	8.9
301007	13,597 (16.10)	7085 (52.11)	6512 (47.89)	1502 (11.06)	11,013 (81.11)	248 (1.83)	815 (6.00)	8.9
301008	12,131 (14.36)	7321 (60.35)	4810 (39.65)	626 (5.16)	11,486 (94.70)	12 (0.10)	5 (0.04)	9.6
301001	11,995 (14.20)	5989 (49.93)	6006 (50.07)	737 (6.16)	10,238 (85.50)	402 (3.36)	597 (4.99)	8.3
301011	5578 (6.60)	2935 (52.62)	2643 (47.38)	413 (7.43)	4735 (85.15)	23 (0.41)	390 (7.01)	9.7
301010	5401 (6.39)	2375 (43.97)	3026 (56.03)	578 (10.73)	4581 (85.05)	16 (0.30)	211 (3.92)	9.2
302000	3982 (4.71)	1928 (48.42)	2054 (51.58)	544 (13.68)	3176 (79.84)	2 (0.05)	256 (6.44)	9.3
301016	2839 (3.36)	1714 (60.37)	1125 (39.63)	165 (5.84)	2574 (91.15)	21 (0.74)	64 (2.27)	8.8
302003	2685 (3.18)	1433 (53.37)	1252 (46.63)	138 (5.16)	2215 (82.77)	51 (1.91)	272 (10.16)	7.8
301029	2342 (2.77)	899 (38.39)	1443 (61.61)	112 (4.79)	1327 (56.81)	27 (1.16)	870 (37.24)	7.3
302001	1293 (1.53)	558 (43.16)	735 (56.84)	79 (6.11)	977 (75.62)	23 (1.78)	213 (16.49)	8.1
502007	1108 (1.31)	432 (38.99)	676 (61.01)	88 (7.96)	943 (85.34)	11 (1.00)	63 (5.70)	8.4
302002	857 (1.01)	470 (54.84)	387 (45.16)	72 (8.41)	657 (76.75)	4 (0.47)	123 (14.37)	9.2
301013	763 (0.90)	313 (41.02)	450 (58.98)	56 (7.38)	629 (82.87)	3 (0.40)	71 (9.35)	11.3
301024	633 (0.75)	288 (45.50)	345 (54.50)	28 (4.50)	439 (70.58)	3 (0.48)	152 (24.44)	8.6
301002	570 (0.67)	356 (62.46)	214 (37.54)	43 (7.54)	521 (91.40)	2 (0.35)	4 (0.70)	9.2
301055	554 (0.66)	186 (33.57)	368 (66.43)	45 (8.12)	491 (88.63)	0 (0)	18 (3.25)	13.5
301054	551 (0.65)	231 (41.92)	320 (58.08)	40 (7.38)	457 (84.32)	2 (0.37)	43 (7.93)	13.6
301050	472 (0.56)	246 (52.12)	226 (47.88)	29 (6.20)	387 (82.79)	26 (5.56)	26 (5.56)	9.0
301063	415 (0.49)	243 (58.55)	172 (41.45)	39 (9.40)	366 (88.19)	0 (0)	10 (2.41)	9.4
301038	197 (0.23)	175 (88.83)	22 (11.17)	8 (4.42)	170 (93.92)	0 (0)	3 (1.66)	8.4
301023	196 (0.23)	68 (34.69)	128 (65.31)	151 (77.44)	14 (7.18)	0 (0)	30 (15.38)	10.2

^a^Represents the mean HbA1c for all registered patients at each health institute.

^b^Health institutes without any impaired glucose tolerance (IGT) cases reflect unavailability of the oral glucose tolerance test (OGTT).

^c^Wide variations in the number of gestational diabetes (GDM) cases reflects unavailability of antenatal care.

**Table 2:** Distribution of consumption and cost of different types of insulin according to diabetes type from the Saudi National Diabetes Registry, 2012 data.

Insulin users and insulin types		Type of diabetes			Total
		Type 1	Type 2	GDM	
Registered patients, n (%)		8058 (26.49)	20,482 (67.34)	1874 (6.16)	30,414 (35.81)
**Regular insulin**					
	Patients, n (%)	2452 (30.94)	4800 (60.56)	674 (8.50)	7926 (26.06)
	Mean units/patient/year^a^	7012	8129	8877	7847
	Total units/year	17,192,566	39,017,040	5,982,963	62,192,569
	Total cost/year (%)^b^	1,602,371 (27.64)	3,636,442 (62.74)	557,620 (9.62)	5,796,434 (14.49)^c^
**Neutral protamine Hagedorn (NPH)**					
	Patients, n (%)	2262 (29.72)	4695 (61.70)	653 (8.58)	7610 (25.02)
	Mean units/patient/year ^a^	9997	12,282	8012	11,237
	Total units/year	22,614,006	57,665,164	5,231,673	85,510,842
	Total cost/year (%)^b^	1,326,365 (26.45)	3,382,199 (67.44)	306,850 (6.12)	5,015,414 (12.54)^c^
**Premixed insulin**					
	Patients, n (%)	1990 (16.70)	9518 (79.89)	406 (3.41)	11,914 (39.17)
	Mean units/patient/year ^a^	18,670	20,433	19,531	20,108
	Total units/year	37,152,803	194,478,439	7,929,647	239,560,888
	Total cost/year (%)^b^	3,435,974 (15.51)	17,985,798 (81.18)	733,351 (3.31)	22,155,123 (55.39)^c^
**Glargine insulin analog**					
	Patients, n (%)	671 (36.04)	1,133 (60.85)	58 (3.11)	1862 (6.12)
	Mean units/patient/year ^a^	9877	9552	8734	9644
	Total units/year	6,627,400	10,822,473	506,598	17,956,471
	Total cost/year (%)^b^	1,988,932 (36.91)	3,247,904 (60.27)	152,034 (2.82)	5,388,870 (13.47)^c^
**Aspart insulin analog**					
	Number of patients (%)	637 (62.39)	304 (29.77)	80 (7.84)	1021 (3.36)
	Mean units/patient/year ^a^	11,895	13,917	12,742	12,564
	Total units/year	7,577,338	4,230,905	1,019,372	12,827,615
	Total cost/year (%)^b^	903,219 (59.07)	504,324 (32.98)	121,509 (7.95)	1,529,052 (3.82)^c^
**Lispro insulin analog**					
	Number of patients (%)	46 (56.79)	32 (39.51)	3 (3.70)	81 (0.27)
	Mean units/patient/year ^a^	11,465	15,593	22,995	13,523
	Total units/year	527,374	498,970	68,985	1,095,329
	Total cost/year (%)^b^	53,673 (48.15)	50,783 (45.55)	7021 (6.30)	111,477 (0.28)^c^
**Cost of insulin therapy**					
	Total patients/year, n(%)	9,310,534 (23.28)	28,807,450 (72.03)	1,878,386 (4.70)	39,996,370 (100)
	Per patient/year (SR)	1155	1406	1002	1315

^a^Mean insulin consumption in units/patients/year.

^b^Percentage of cost/year for each insulin type for different diabetes types. Cost is calculated in Saudi Riyals (SR), in which US $1=3.75 SR.

^c^Percentage of each insulin type cost in reference to the total insulin cost.

### Geographic Reports

**Figure 3:**
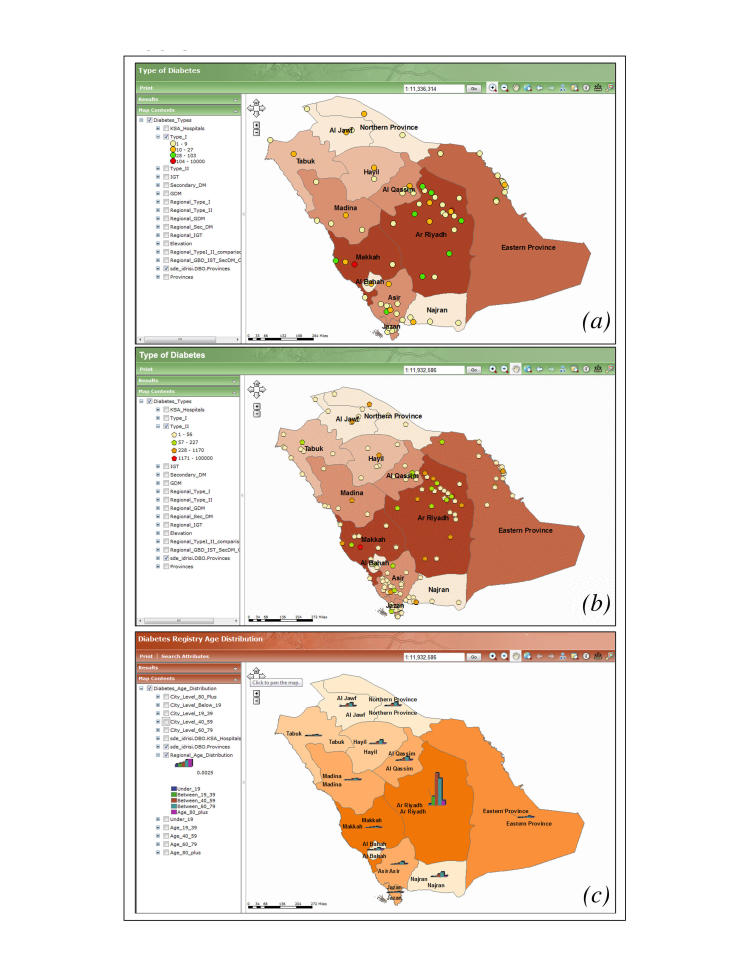
Geographic information system (GIS) maps demonstrating the diabetic patient distribution for (a) type 1 diabetes and (b) type 2 diabetes at the country level, and (c) the distribution of different age groups in all health sectors.

**Figure 4:**
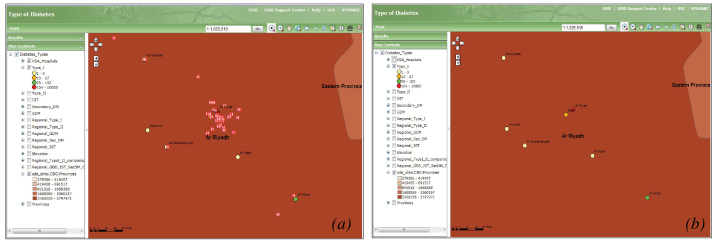
Distributions of (a) hospital (H) locations and (b) type 1 diabetes patients living in Ar Riyadh. The projection scale for these maps is 1:1,029,519.

The SNDR has a designed function to provide a variety of maps for any covariates. Figure 3 shows examples of GIS map screenshots for major diabetes types and age distribution in the Kingdom. The map in part (a) shows a GIS screenshot of type 1 diabetic patients’ distribution in the entire country represented by the total number of registered cases. This shows a larger number of type 1 diabetic patients located in the major cities. Part (b) shows type 2 diabetic patients’ distribution in different health regions according to the total number of patients that show more distribution in medium-sized cities, in addition to large cities compared with villages and rural areas. Part (c) is a histogram of different age groups in different heath regions. The age groups 40 to 59 years and 60 to 79 years represent the highest percentage distribution in almost all health regions. A magnified version of the type 1 maps, showing the cities of patient’s residence along with the distribution of hospitals, is provided in Figure 4.

The gradation on the maps is a representation of the Saudi population at the regional level. Riyadh, being one of the most densely populated regions, has the darkest gradation. Point symbology is a representation of patients registered from various cities of the Kingdom. Colors of the points are the representation of the total count of patients registered from that particular city. The symbol H represents a hospital.

**Figure 5:**
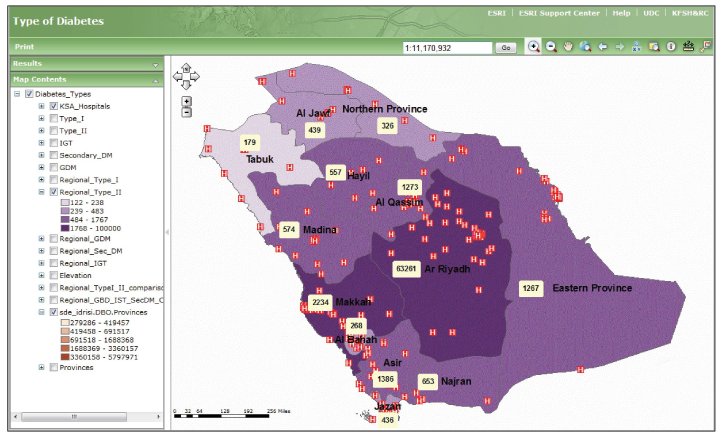
Regional distribution of type 2 patients and hospital (H) locations across the Kingdom. The projection scale for this map is 1:11,170,932.

Figure 5 provides regional patient counts for type 2 plus distribution of hospitals around the Kingdom.

The reason for including hospitals, along with a distribution of patients, was to determine the availability of health care facilities required for the targeted treatment and to ensure the communication with the Ministry of Health for provision of the required resources.

### Research Tool

**Figure 6:**
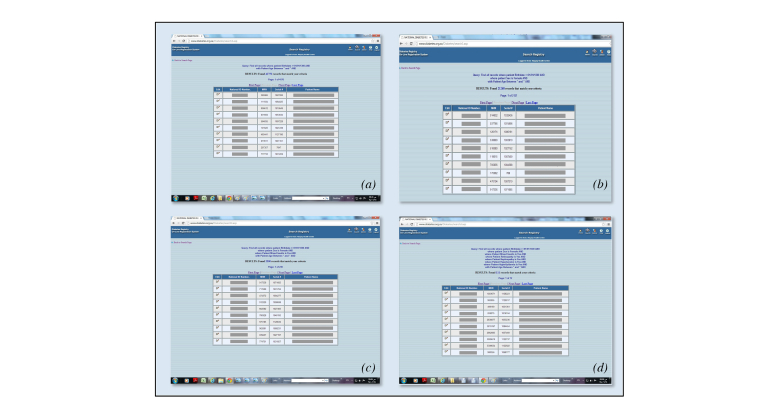
Lists of patient names, national IDs, and hospital and serial numbers generated by the search tool of the SNDR at different stages: (a) all patients between ages 45-65 years, (b) who are female, (c) using premixed insulin, (d) with proliferative retinopathy, microalbuminuria, hypertension, and hyperlipidemia.

The SNDR has a wide range of search options by using different parameters and geographic choices. Figure 6 demonstrates examples of the search facilities, each providing a list of patient names, national IDs, hospital medical numbers, and registry serial numbers that can give direct access to the patients’ medical files. In Figure 6, a total of 41,572 patients were found when searching for patients aged between 45 and 65 years, of which 21,268 were women. Of these female patients, 2300 were on premixed insulin only; of these on premixed insulin, 111 patients were found with proliferative retinopathy, microalbuminuria, hypertension, and hyperlipidemia.

### Health Care Planning Report

**Figure 7:**
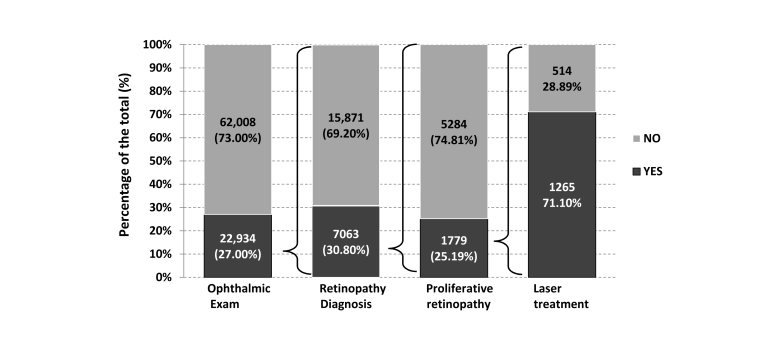
The ophthalmic exam coverage and retinopathy diagnosed patients with proliferative retinopathy and those who received or missed laser therapy among the total registered patients.

The SNDR is a very useful tool for assessing health care systems and providing advice for future planning. Figure 7 uses ophthalmic examination as an example to test this system in which 84,942 patients’ data were analyzed for fundus eye evaluation for retinopathy. Only 22,934 (27.00%) of those registered cases had an ophthalmic exam, of which 7063 (30.79%) were found to have retinopathy. Of all cases with retinopathy, 1779 (25.18%) had proliferative retinopathy warranting laser therapy. Only 1265 (71.10%) had laser therapy; 28.89% did not have access or refused this therapy. Applying the same percentages, of the 62,008 (73.00%) patients who did not have the fundus examination, 19,098 would be expected to have retinopathy, out of which 4812 would need laser therapy.

## Discussion

The SNDR is unique in its functionalities, which are not found in other diabetes registries that either risk stratification, such as Joslin’s Web-based Diabetes Registry and Risk Stratification System [20], or track clinical outcome, such as the PSHDOI [21], or test embedded guidelines for chronic diseases, such as the CDEMS [22]. No other registry is available today that has similar functionalities and interaction designed for live data queries from the registry. This is the first of its kind to implement GIS as a query layer for a diabetes database, and has linkages to the live governmental citizen ascertainment database.

This descriptive study is the first report to test the functionalities after 12 years of development. The registry’s current annual growth rate of 10% gives hope that most diabetic patients will be registered in the Kingdom in less than 10 years. The male to female ratio and different diabetes types are identical to what has been shown internationally. Gender distribution demonstrates the predominance of male gender, whereas the distribution of diabetes types is similar to what is known internationally [28]. The IGT cases were underestimated at the beginning of the registry in the year 2000, but more cases have been reported by the year 2012, which could reflect better case screening or the involvement of primary care centers, which are more likely to catch such cases. However, IGT cases are still underestimated when compared with the expected number of cases in the Kingdom [12].

The SNDR provides epidemiological indicators, such as incidence, prevalence, mean, and median, that will help to better understand and plan for this disease management. The 22 randomly selected hospitals show variability in gender and diabetes types, which reflect the nature of the hospital (eg, general, pediatrics, or maternity), whereas patient number variations reflect the hospital bed count and outpatient services. The large discrepancies in the distribution of diabetes types can be explained by their diabetic population related to service availability. The mean HbA1c variation is a reflection of the health care management provided by those hospitals.

The SNDR is the first of its kind to provide health care planners with an instantaneous and accurate cost estimation of different aspects related to diabetes and its management. The registry system is capable of estimating both direct and indirect diabetes costs using diabetic population data and local services costs. The annual insulin cost used by the insulin-treated patients in the registry demonstrated that type 2 diabetics are the most frequent insulin users, secondary to the large number of patients and higher doses used per patient, similar to what was shown by Tomlin et al [29]. This confirms that the annual insulin cost is higher in type 2 diabetic patients than type 1 or gestational diabetes, similar to Kumamoto’s study conducted in Japan in the year 2000 [30]. Premixed insulin, mostly delivered by insulin pen, consumed more than half of the total insulin cost, whereas mixing regular and NPH by syringes made up 12.54% and 14.49% of the cost. Fewer insulin analogs were used because of their limited availability in the Ministry of Health health care facilities.

The GIS mapping for diabetes types, risk factors, and complications in different health regions in relation to environmental or municipal variables shed further light on the relationship between this disease and different environmental factors. Mapping type 1 diabetes distribution showed that more patients were located in larger cities. This could be because they are more populated than smaller cities, and have specialized health care facilities for this type of diabetes. The higher aggregation of type 2 diabetic patients in medium-sized cities is a reflection of its high prevalence and a wider distribution of primary care centers that provide health care for such patients when compared to smaller cities. The SNDR geographic mapping with its statistical power can produce any countrywide clinical and nonclinical variables that can compare data from different health regions. This study has used age to map different groups in defined geographic areas. When examining the age group 45 to 65 years for the prevalence of diabetes, the result was consistent with local and international epidemiology studies that have found the highest concentration of diabetes among this population [31,32]. The GIS system, as shown, provides a link between the clinical data and health care facilities availability around the patient’s location.

By using SNDR as a research tool, it can provide answers for queries related to registry data that will cover medical, social, and cultural parameters. It can provide researchers with patient lists related to specific inclusion and exclusion criteria, and give clinical details for the selected samples and provide answers for the research query. Because of the large number of registered patients, there is sufficient sample strength available to study even with the toughest inclusion and exclusion criteria. As shown in this study, selecting females aged between 45 and 65 years, using only premixed insulin, diagnosed with proliferative retinopathy with microalbuminuria, hypertension, and hyperlipidemia, yielded 111 patients, which is enough to conduct any retrospective or prospective studies.

To test the SNDR as a tool to investigate health care facilities and practice, retinal examination and laser therapy were used to assess medical services available in different health regions. The ophthalmic examination data available revealed that there was underscreening of more than 70% of diabetic patients and one-third of the patients who needed laser therapy did not get it either because it was not available or because of patients’ misconception about this therapy. It is expected that one-quarter of the unscreened patients will need laser therapy, and the patients’ vision may be threaten if not done. These findings give health planners the chance to discover such problems and give the right advice to overcome any obstacles in treating or preventing diabetic complications.

In conclusion, the SNDR as a data bank for diabetic patients’ medical files is useful in monitoring the disease and its chronic complications or associated diseases. The developed epidemiology, economy, or geographic reporting system used in the SNDR is practical, useful, and accurate in assessing and forecasting this chronic disease monitoring and management. The SNDR reports provide health care planners, researchers, and governmental departments with data needed to understand this disease and will allow the launching of primary and secondary prevention programs that could reduce the size of the problem and its economic burden.

## Abbreviations

ADA: American Diabetes Association
CDEMS: Chronic Disease Electronic Management System
GCS: geographic coordinate system
GDM: gestational diabetes mellitus
GIS: geographic information system
HDL: high-density lipoprotein
IDF: International Diabetes Federation
IGT: impaired glucose tolerance
LDL: low-density lipoprotein
NPH: neutral protamine Hagedorn
OGTT: oral glucose tolerance test
PSHDOI: Penn State Hershey Diabetes and Obesity Institute
SGOT: serum glutamic oxaloacetic transaminase
SGPT: serum glutamic-pyruvic transaminase
SNDR: Saudi National Diabetes Registry
SR: Saudi Riyals
WHO: World Health Organization
